# Geometrical Conditions Indispensable for Muscle Contraction

**DOI:** 10.3390/ijms12042138

**Published:** 2011-03-29

**Authors:** Ludmila Skubiszak

**Affiliations:** Nałęcz Institute of Biocybernetics and Biomedical Engineering of the Polish Academy of Sciences, Ks. Trojdena 4, 02-109 Warszawa, Poland; E-Mail: lskubiszak@ibib.waw.pl; Tel.: +48-22-6599143; Fax: +48-22-6597030

**Keywords:** muscle contraction mechanism, force generation, splitting of the M3 reflection, intensity increasing of the M15 reflection, muscle sarcomere, computer simulation

## Abstract

Computer simulation has uncovered the geometrical conditions under which the vertebrate striated muscle sarcomere can contract. First, all thick filaments should have identical structure, namely: three myosin cross-bridges, building a crown, should be aligned at angles of 0°, 120°, 180°, and the successive crowns and the two filament halves should be turned around 120°. Second, all thick filaments should act simultaneously. Third, coordination in action of the myosin cross-bridges should exist, namely: the three cross-bridges of a crown should act simultaneously and the cross-bridge crowns axially 43 and 14.333 nm apart should act, respectively, simultaneously and with a phase shift. Fifth, six thin filaments surrounding the thick filament should be turned around 180° to each other in each sarcomere half. Sixth, thin filaments should be oppositely oriented in relation to the sarcomere middle. Finally, the structure of each of the thin filaments should change in consequence of strong interaction with myosin heads, namely: the axial distance and the angular alignment between neighboring actin monomers should be, respectively, 2.867 nm and 168° instead of 2.75 nm and 166.15°. These conditions ensure the stereo-specific interaction between actin and myosin and good agreement with the data gathered by electron microscopy and X-ray diffraction methods. The results suggest that the force is generated not only by the myosin cross-bridges but also by the thin filaments; the former acts by cyclical unwrapping and wrapping the thick filament backbone, and the latter byelongation.

## 1. Introduction

It is well established that the vertebrate striated muscle contracts due to shortening of its quasi-cells, sarcomeres [1–3]. Sarcomere contraction is coupled with mutual sliding of the two kinds of filaments, thick (myosin-based) and thin (actin-based). The filaments are arranged into bipolar hexagonal lattice, in each half of which the thick filament is surrounded by six thin ones and each thin filament by three thick ones [4–7]. In the relaxed state, the myofilaments occur as individual rods, but during contraction, they are connected by myosin cross-bridges [8–10]. Tension generated during contraction is proportional to the sarcomere length [11], as well as to the number of the cross-bridges [12]. Actin-myosin interaction is a prerequisite of muscle contraction [13–15]. The interaction is stereo-specific because it is possible only after precise hitting of actin binding-site specific for each myosin head into myosin binding-site specific for each actin monomer [16–19]. The dimensions of each binding-site are much smaller than the dimensions of either actin monomer or myosin head. Actin-myosin interaction is closely coupled with ATP hydrolysis. The hydrolysis occurs within the myosin head and triggers its conformational changes [18,20–23]; a head without nucleotide has the straight configuration while it is bent with ADP. So, for a sarcomere contraction, myosin cross-bridges must move from the thick filament surface towards the surrounding thin filaments in such a way to enable the precise hitting. The way of cross-bridge movement is the most controversial aspect of muscle contraction because none of the available experimental methods can directly visualize a separate molecule within the thick filament.

Structure of myosin molecule is quite well established by *in vitro* experiments (reviewed e.g., in [24,25]); it is a long tail ended by two globules, called the heads. The tail contains two coiled-coil α-helices; the head has quite a complex atomic structure [18,20]. It is clear that the manner of cross-bridge action depends on the arrangement of myosin molecules within thick filament. For over forty years, the vertebrate striated thick filament has been described as a three-stranded rod consisting of myosin molecules arranged parallel or nearly parallel to the filament axis (reviewed e.g., in [26–29]). In consequence, the myosin cross-bridge action is described in an oar-like manner, *i.e*., by bending the molecule within three hinge domains: one localized within the tail, second in the place where the coiled-coil myosin tail untwists into the two heads, and third within the head. After discovery of the two conformations of myosin head [18,20–22], the so-called swinging lever-arm [30] or tilting lever-arm [31], mechanism of force generation was introduced. According to this concept, the force is generated due to rotation of the lever-arm domain of myosin head, linked with the myosin tail, in relation to its motor domain, connected to the actin monomer (reviewed e.g., in [32,33]). The rotation is considered to be caused by the stereo-specific interaction with actin monomer and coupled with the ATP hydrolysis.

In this work, a new action of myosin cross-bridges is considered, namely, by cyclical unwrapping and wrapping the filament backbone. Such action follows from twisted arrangement of myosin tails, previously introduced [24,25] as the only way for reconstruction of the real features of the vertebrate striated thick filament, first of all, its bipolar tube appearance.

The two radically different concepts of myosin cross-bridge action are analyzed in the work by computer simulation of mutual sliding of the two kinds of filaments. The approach gives a unique possibility to address the following problems: (1) to select the structure that ensures optimal stereo-specific interaction between actin and myosin; (2) to verify thin filament structure during contraction; (3) to arrange the two kinds of filaments within the bipolar hexagonal lattice; (4) to find the correlation in cyclical action of myosin cross-bridges. The geometrical conditions, established using our simulation method, cannot be directly confirmed by the available experimental methods. They are justified, as is usually accepted, by comparison of the calculated Fourier spectra with actual diffraction patterns available in literature.

**Figure 1.**
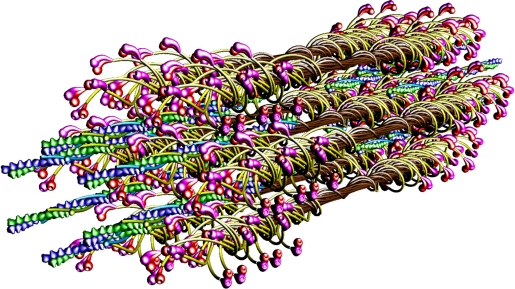
Molecular structure of the vertebrate striated muscle sarcomere. Seven myosin filaments and twelve thin filaments are shown during a phase of contraction. The myosin cross-bridges belonging to one crown and those belonging to the crowns axially distanced by 43 nm are identically unwrapped, and the cross-bridges belonging to three successive crowns are unwrapped by 0.9; 0.95; 0.0. Each of the seven myosin filaments has the structure denoted in the previous work [53] by 1L-1L/120. The brown and yellow rods depict two subfragments of myosin tails: the light meromyosin (LMM) and the subfragment 2 (S2), respectively. The two myosin heads are shown as magenta and red globules. The hollow at each head represents the actin binding-site. Each of the twelve thin filaments has the helical structure in which the neighboring actin monomers are axially 2.867 nm apart and are turned around 168°. Each actin monomer is computed as a ball covered by a cone; the cone depicts myosin binding-site. Successive actin monomers are shown in different colors, thereby, a double-stranded right-handed superhelix is noticeable. The tropomyosin molecule is presented as a blue or green ribbon, and the troponin molecule as a blue or green globule. In each sarcomere half, the six thin filaments surrounding the central thick filament are turned around 180°, and the thin filaments in two halves are oppositely oriented in relation to the sarcomere centre.

The molecular structure of the vertebrate striated muscle sarcomere, presented in Figure 1, allows interpreting such specificities of the diffraction patterns as: (1) arrangement of all reflections along the layer lines distanced by 14.33 nm; (2) appearance of the so-called forbidden reflections; (3) splitting of the M3 reflection; (4) intensity increasing of the M15 reflection. The splitting of meridional reflection at third layer-line, *i.e*., the M3 reflection, observed in diffraction patterns from living muscle [34–36] is presently quoted [23,31,33,37] to be the argument for correctness of the tilting lever-arm mechanism. It is interpreted that the splitting reflects the two configurations of myosin head [20,38]: straight and bent. However, the mechanism does not seem to be convincingly argued. First of all, it is usually discussed by consideration of one myosin head and a fragment of actin filament [23,33,39]; therefore, the question of the precise hitting between hundreds of binding-sites within the bipolar hexagonal lattice of the contracting sarcomere is beyond our understanding. Secondly, the degree of rotation of the natural head has not been determined; the rotation quoted by different authors [40–42] for the modified heads ranges from a few up to about 160°. Finally, the conformation of myosin head, connected either with actin monomer or/and nucleotide, is unknown.

## 2. Results and Discussion

Computation of the hexagonal lattice of vertebrate striated muscle sarcomere from different number of elements and calculation for each of the modes of the Fourier spectrum give a unique possibility to investigate the relation between sarcomere structure and specificity of spectrum. The approach used in this work is justified because values of all parameters are related to real dimensions gathered in literature. It is worth noting, however, that the real dimensions are usually given with some range; so, the values were checked to be within the range.

The actual spectra [34–36,43–52] reveal the following specificities: (1) the distribution of reflections along separate layer-lines distanced by 1/14.333 nm^−1^; (2) the appearance of prominent reflections along the meridian; (3) the splitting of the M3 reflection; (4) the intensity increasing of the meridional reflection at the fifteen layer line (usually termed the M15 reflection). The two first features are specific for the both physiological conditions, relaxed and contraction; the features 3 and 4 occur in transition from the relaxed state into contraction.

**Figure 2.**
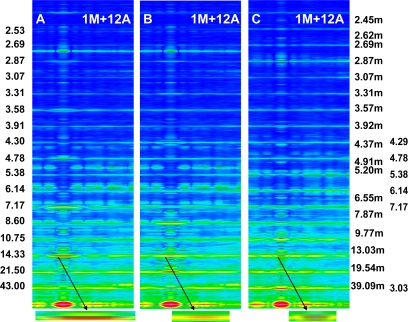
Fourier spectra calculated for the bipolar hexagonal lattice containing one myosin filament, denoted by 1M, surrounded by twelve thin filaments, denoted by 12A. (**A**) All myosin cross-bridges are identically wrapped around the myosin filament backbone; in consequence, all myosin heads are on the filament surface and the cross-bridge crowns are axially 14.333 nm apart; (**B**) The myosin cross-bridges belonging to the same crown as well as to the crowns axially distanced by 43 nm are identically wrapped, and the myosin cross-bridges belonging to three successive crowns are unwrapped by 0.25, 0.15, 0.05; in consequence, all myosin heads are nearly on surface, but the axial distances between the crowns are slightly different: 14.67, 14.66, and 13.67 nm; (**C**) The myosin cross-bridges belonging to three successive crowns are unwrapped by 0.9, 0.95, and 0.0; in consequence: (1) 2/3 of myosin heads are in the vicinity of thin filaments and 1/3 on the thick filament surface, and (2) the axial distances between the crowns are 13.00, 1.66, and 28.67 nm. To the left and right, the values of repeats specific for, respectively, myosin and thin filaments, are shown which are detected by the successive layer lines. In the case of thin filaments, the number with “m” corresponds to the reflection arranged along the meridian; the numbers without “m” correspond to the off-meridional reflections. Further down, the zoomed M3 reflections are shown.

The Fourier spectra calculated for the two specific states (Figure 2) clearly demonstrate good consistency with the experimental data. In the work, the spectra are presented for the sarcomere computed on the basis of myosin filament previously denoted by 1L-1L/120[53], but the filaments computed on the basis of remaining crown configurations, *i.e*., 1M, 2L, 2M, possess the same features.

The living muscles are usually investigated by the low-angle X-ray diffraction patterns. The method detects mostly arrangement of myosin heads, actin monomers and troponin heads because the volume and mass of each of them are bigger than those of the remaining elements. The reflections specific for thin filaments are less prominent than for thick filaments. The intensity of reflections depends on the number of elements taken into consideration.

**Figure 3.**
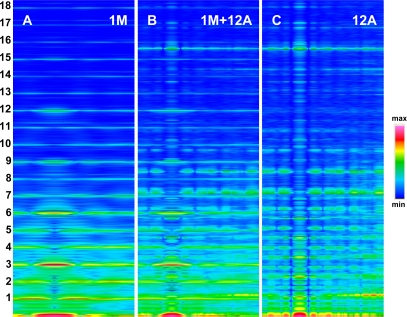
Fourier spectra calculated for one myosin filament, denoted by 1M (**A**), twelve thin filaments, denoted by 12A (**C**), and for one myosin filament surrounded by twelve thin filaments, denoted by 1M + 12A (**B**). The sarcomere structure is simulated for relaxed state, *i.e.*, all myosin cross-bridges are identically wrapped around the thick filament backbone, and in thin filament, the axial distance and the rotation between neighboring actin monomers are 2.75 nm and 166.15°, respectively. To the left, the successive layer lines specific for the spectrum of thick filament are numerated. The layer lines specific for the spectrum of thin filament are shown in Figure 2. To the right, a scale of the relative intensities of reflections is shown.

**Figure 4.**
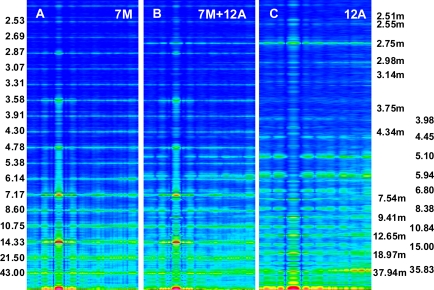
Fourier spectra calculated for seven myosin filaments, denoted by 7M (**A**), twelve thin filaments, denoted by 12A (**C**), and for the two kinds of filaments arranged into bipolar hexagonal lattice, denoted by 7M + 12A (**B**). The values of used parameters are the same as in the case of Figure 3. The numbers shown to the left and right have the same meaning as in Figure 2.

The foregoing relations are clearly revealed in Figures 3A and 4A. The reflections are similarly arranged because the distribution of all elements is similar in the both cases. But the spectrum for one myosin filament (Figure 3A) differs from that of seven others arranged into bipolar hexagonal lattice (Figure 4A); the difference is caused by interference within the bipolar hexagonal lattice. The spectra in Figures 3B and 4B demonstrate the influence of the two kinds of filaments; in the former, the contribution of thin filaments is more distinct than in the latter.

The resulting relations justify the approach used in the work for verification by comparison of the spectrum specificities, not by comparison of the reflection intensities, as is commonly believed to be the case.

### 2.1. Layer-line Character of the Spectrum

The presented spectra clearly demonstrate that the layer-line character of the spectrum is determined by grouping of myosin heads into separate crowns. The distance between the layer-lines along the meridian is 14.333 nm if the crowns are axially 14.333 nm apart. The prominent meridional reflections appear at the third, sixth, ninth, *etc*., layer-lines, if: (1) the projection of mass density on a plane parallel to the filament axis from each of the three successive crowns is different, but from the crowns axially distanced by 43 nm is identical; (2) all myosin filaments are identically arranged and act simultaneously.

### 2.2. Forbidden Reflections

The meridional reflections are usually denoted by M1, M2, M3, *etc*., because the reflections are determined by arrangement of the myosin heads. The vertebrate striated muscle thick filament is commonly considered either as a three-stranded rope of a 3 × 43-nm pitch [54] or as a cylinder covered by the myosin heads arranged along three symmetrically originated helical paths of the 3 × 43 nm pitch [55]. Thereby, in the relaxed state, the meridional reflections are expected at the M3, M6, M9, M12, *etc.*, layer lines, *i.e*., at the lines corresponding to 3n order of the 43-nm repeat. The expected feature does not agree, however, with the actual spectra, in which the meridional reflections occur at all layer lines. Thereby, the reflections M1, M2, M4, M5, M7, *etc*., are usually called “forbidden” for the 9/3 helical symmetry and are interpreted by perturbations in arrangement of the myosin heads on the filament surface [44,47,48,56–58]. From another viewpoint, the reflection M3, corresponding to 14.333-nm axial repeat, is usually interpreted [23,31,32,37,59] as depending on the projection of mass density of myosin heads onto a plane parallel to the filament axis.

Previously [53] demonstrated that virtually ideal consistency of the thick filament features reconstructed and established experimentally can be obtained for twelve bipolar thick filaments. These filaments are identical from the point of view of both the myosin tail arrangement and the myosin head grouping; the tails are twisted and the crowns are symmetrical. Such filaments do not look like a three-stranded rope. The structure of each is more complex; *i.e.*, a tube covered by myosin heads (Figure 1). The distribution of myosin heads on the filament surface cannot be precisely described by three symmetrically originated helical paths of the 3 × 43 nm pitch. Nevertheless, all twelve filaments reveal the correct, three-fold rotational symmetry despite each of the crowns is asymmetrical (Figures 4, 7 in [53]). The meridional reflections occur only at the layer-lines M3, M6, M9, *etc.*, (Figures 3A,B, 4A,B) if all myosin cross-bridges are identically wrapped around the filament backbone. The meridional reflections occur at all layer-lines (Figures 2B,C, 6A,B), *i.e*., also at the so-called “forbidden” for the 9/3 helical symmetry if the myosin cross-bridges belonging to the same crown are identically wrapped, and those belonging to three successive crowns differently. It is worth noting, that appearance of the meridional reflections at all layer lines occurs even after introduction of a slight difference in axial distances between three successive crowns (compare Figure 2A,B). The difference determines the relative intensities; the most prominent being the reflections M2 (see Figure 2B) or M1, not M3.

The filament reconstructed on the basis of symmetrical crowns, denoted by 4D-4D reveals a similar relationship between the arrangements of both the cross-bridge crowns along the filament axis and the appearance of meridional reflections (Figures 6, 8 in [53]). But the relation between symmetries of the whole filament and each of the crowns is radically different than in the case of the filaments considered above; the filament has nearly cylindrical symmetry and each of its crowns has the three-fold rotational symmetry (Figures 4, 7 in [53]). The distribution of myosin heads on the filament surface can be, in the case of this filament, precisely described by three symmetrically originated helices of the pitch 3 × 43nm (Figure 5 in [53]).

The analysis clearly demonstrates that arrangement of the meridional reflections does not depend on symmetry of the thick filament, as is commonly believed; it detects the arrangement of the cross-bridge crowns along the filament axis. Moreover, the identical unwrapping simulates the simultaneous action of myosin cross-bridges, and different unwrapping—a phase shift in action of the neighboring crowns. Thereby, we can conclude that the arrangement of meridional reflections exposes coordination in action the myosin cross-bridges; thus, none of the reflections should be considered as forbidden.

### 2.3. Splitting of the M3 Reflection

Splitting of the M3 reflection is observed in the spectra from living muscle in conditions of transition from relaxed state into contraction [34–36]. The splitting is differently interpreted in literature [23,31,33,37], but commonly as a strong argument for correctness of the tilting lever-arm mechanism of force generation [23,32,60,61]. It is clear that the projection of head density on a plane parallel to the filament axis depends on the head orientation in relation to the plane. So, the occurrence of splitting is possible if the head moves either in the way commonly accepted, *i.e*., by bending on a plane between the thick and thin filaments, or in the way proposed in the work, *i.e*., by unwrapping and wrapping the filament backbone. Simulation of the sarcomere contraction by different arrangements of myosin heads in 3D space of the sarcomere has given a possibility to analyze the conditions under which the splitting can occur.

The analysis clearly demonstrates that the head configuration should not be considered as needful effect responsible for the distinct splitting; the splitting occurs if the two heads are bent (see row 10 in Figure 6) as well as if they are straight (see row 6 in Figure 6). The splitting does not also depend on the mutual alignment of the two heads (compare the rows 1 with 10 in Figure 6) as well as on the configuration of myosin cross-bridge crowns (compare the rows 1 with 11 and 12 in Figure 6).

The computer simulation has allowed establishing of five conditions which should be simultaneously fulfilled. First, the bipolar structure of thick filament must be taken into consideration (the rows 1 and 2 in Figure 6). This result agrees with the interpretation made by Linari and co-workers [36] that the splitting is a consequence of interference from the two halves of thick filament. Second, the two heads of the same myosin molecule must be taken into consideration (compare the rows 1 with 3 and 4 in Figure 6). Third, the two heads of the same myosin molecule must be slightly spaced (compare the rows 1 with 5 and 6, as well as with 7 in Figure 6). Forth, the rotation between the left and right halves of the filament must be 120° or 90° (see Figure 7). Finally, the three successive cross-bridge crowns must act with a specific phase shift (compare the rows 1 with 8 in Figure 6, as well as see Figure 8C,D,I,J). The condition that the two sets of cross-bridges of slightly different configurations should exist (see the rows 1 and 8 in Figure 6, as well as Figure 7C,D,I,J) confirms the interpretation made by Bordas and co-workers [34]. The configurations denoted by 0.9 and 0.95 means that the cross-bridges belonging to two neighboring crowns are nearly at the same distance from the surrounding thin filaments, and the configuration 0.0 depicts the cross-bridge localization on the thick filament surface. In consequence, two of three cross-bridge crowns are very close, about 1 nm apart. The results suggest that the three successive cross-bridge crowns should act with a specific phase shift.

### 2.4. Intensity Increasing of the M15 Reflection

**Figure 5.**
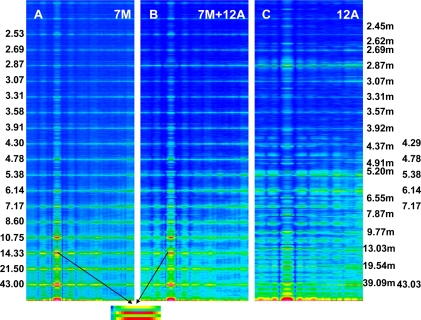
Fourier spectra calculated for the conditions of contraction. In (**A**), seven myosin filaments, in (**C**), twelve thin filaments, and in (**B**), the two kinds of filaments arranged into bipolar hexagonal lattice are shown. The numbers to the left and right have the same meaning as in Figure 4.

Intensity increasing of the M15 reflection in the spectrum from contracting muscle in comparison to the spectrum from relaxed muscle observed for the living muscle [31,49–51,56] is usually interpreted by compliance of the thick filament; the F-actin filament helix is usually regarded as a rigid rod [10]. Calculation of the spectra for the two conditions, *i.e.*, for relaxed state (Figure 2A) and for one of the contraction phases (Figure 2C) as well as separately for the myosin filaments (Figure 5A) and for the thin filaments (Figure 5C) has allowed to clearly demonstrate that the intensity increasing of the M15 reflection is caused by the introduced elongation of the thin filament. The contribution of thin filament structure is expressed by consideration of one myosin filament instead of seven ones (compare Figures 2C and 5B). In the case of relax, the M15 reflection, corresponding to 2.867 nm, is much weaker than the reflection corresponding to the 2.75-nm axial distance between the neighboring actin monomers (see Figures 3B and 4B). In the case of contraction, the distinct reflections at the layer line corresponding to 2.867 nm as well as of the off-meridional reflections at the 1st, 2nd, 3rd, 4th, 5th, and 6th layer lines occur (compare Figure 2A,C). This result agrees well with that observed in result of transition from relaxed state into contraction [49–52,56] and can be considered as a strong argument for fitting of the thin filament structure to arrangement of the myosin cross-bridges in the sarcomere space.

Consideration of the seven myosin filaments (Figure 5B) instead of one (Figure 2C) allows exposition of the contribution of myosin heads on the sarcomere spectrum; the intensity of meridional reflections, including the splitting of the M3, are more distinct in Figure 5B than in Figure 2C. *Vice versa*, the contribution of the thin filament on the spectrum becomes prominent if one myosin filament is taken into consideration (compare Figure 5B with Figure 2C or Figure 4B with Figure 3B).

### 2.5. Conditions under Which the Stereo-Specific Interaction Is Possible

In light of newly available experimental evidence, it seems reasonable to presume that the geometrical conditions indispensable for the sarcomere contraction are identical with those under which the stereo-specific interaction between actin and myosin can occur. Moreover, it seems reasonable to presume that all myosin cross-bridges which reach the surrounding thin filaments should be capable to strong connection with proper actin monomers. Such geometrical conditions could be called optimal.

Simulation of sliding of the two kinds of filaments and observation of the needful hitting between the specific binding-sites has revealed that the optimal hitting is possible (Figure 8) only if: (1) all myosin filaments have the structure denoted by 1L-1L/120; (2) all myosin filaments are identically oriented within the hexagonal lattice; (3) all myosin filaments act synchronously; (4) there is a strong coordination in action of the myosin cross-bridges; (5) the structure of each of the thin filaments changes; (6) the six thin filaments are arranged around each myosin filament by rotation at angle of 180°; (7) the thin filaments are oppositely oriented in the two polar halves. The conditions ensure that one of two myosin heads of each of three cross-bridges which reach the proper thin filament hits into the proper actin monomer with the precision 0.5 nm (Figure 8B); in consequence, the strong interaction is possible.

In the filament 1L-1L/120 (the condition 1), the three pairs of myosin heads are aligned in each crown at angles of 0°, 120°, 180°, and the successive crowns as well as the two filament halves are turned around 120°. The coordination in action of the myosin cross-bridges (condition 4) should be following (Figure 8A): (1) the cross-bridges belonging to the same crown as well as to the crowns axially distanced by 43 nm should be in the vicinity of surrounding thin filaments; (2) after 14.333 nm sliding of the two kinds of filaments, similar set of the cross-bridges, axially distanced from the first set by 14.333 nm, should be in the vicinity of surrounding thin filaments; (3) after next 14.333 nm sliding, the third set of the cross-bridges, axially distanced from the second set by 14.333 nm, should be in the vicinity of surrounding thin filaments. The resulting coordination in action of the cross-bridges is identical with that determined on the basis of the M3 splitting. In consequence of the strong interaction with myosin head, the alignment and the axial distance between the neighboring actin monomers within the genetic helix should change (condition 5), respectively, from 166.15° to 168° and from 2.75 to 2.867 nm. The new parameters ensure the precise hitting because each fifth actin monomer is correctly oriented in relation to three surrounding myosin filaments: 24° × 5 = 120°, and 2.8666 × 5 = 14.333 nm. Animation of the filament sliding is presented at site http://sarcomere.ibib.waw.pl.

The remaining four configurations of the myosin cross-bridge crowns, *i.e*., 1M, 2L, 2M, and 4D, differ from the 1L configuration by alignment of the three pairs of myosin heads within the crown and by axial rotation between the successive crowns. The filaments computed on the basis of either 1M or 2M crown configurations display the hitting only for two of the three cross-bridges protruding from the thick filament backbone at the same level. Moreover, in the case of these configurations, any simultaneous hitting is impossible in the two halves of the bipolar hexagonal lattice after each 28.67 nm shortening of the sarcomere. In the case of the filament 2L-2L, the precise hitting is possible in the both halves simultaneously, but only for two of the three cross-bridges.

The commonly accepted configuration, 4D, reveals very rare hitting between a pair of the two kinds of filaments; within the bipolar hexagonal lattice the hitting is practically impossible.

## 3. Method

Particular phases of muscle contraction are simulated by a specific localization of the myosin cross-bridges in the space between the two kinds of filaments and different degrees of overlapping of the two kinds of filaments, myosin and thin ones. The cross-bridge is depicted by two heads tethered by the S2. The localization of each myosin cross-bridge is manipulated by unwrapping of the S2 from the filament backbone built from the LMMs (Figures 1, 8B). The myosin cross-bridge position on the thick filament surface is assigned by number 0, and when the cross-bridge is on the thin filament surface—by number 1. All intermediate localizations are assigned by numbers from 0 to 1. The program allows monitoring the hitting between the binding-sites with different precision. It is presumed that after each 14.333 nm sliding of the two kinds of filaments all myosin cross-bridges which reach the surrounding thin filaments must hit the proper actin monomers with a spatial precision 0.5 nm. Such hitting, called optimal, is automatically registered in the analytical way (by drawing up a table) and in the graphic way (the proper globules switch on, Figure 8).

The parameters used for reconstruction of all elements as well as of their alignment in the 3D space of hexagonal lattice (Figures 1, 8) are the same as those introduced previously [53]). Each myosin filament is reconstructed from individual subfragments of myosin molecule, *i.e*., from the LMM of 100.7 nm in length, the S2 of 62 nm in length and the two heads. The diameter of the myosin tail, consisting of the LMM and S2, is 2 nm. The shape and volume of the myosin head were introduced and verified previously [62] by superimposing on the structure found by Rayment and co-workers [63]; its lengths in straight and bent configurations are 19 and 16.5 nm, respectively. The localization of the myosin binding-site within the head, depicted by a hollow, was specified in the same way.

The framework of thin filament, usually called the F-actin filament, is reconstructed in accordance with the present-day view (reviewed in [64]), *i.e*., it consists from of the G-actin monomers arranged into a single left-handed genetic helix in which each monomer is related to the next by a rotation of 166.15° around the axis and by an axial translation of 2.75 nm. The thin filament during contraction is constructed on the basis of 2.867-nm axial translation and 168° rotation between the neighboring actin monomers. The actin monomer is depicted by a ball of 5.5 nm in diameter covered by a cone of 0.2 nm in height; the cone depicts the myosin binding-site. Along two gaps between the two strands of the actin helix, the tropomyosin-troponin (Tm-Tn) complexes are arranged. The long Tm molecules, each depicted as a flexible rope, 2 nm in diameter and 40.6 nm in length, are jointed into ribbons. The Tn molecule is computed as a slightly elongated ellipsoid (4.0 × 4.5 nm) tilted at about 75° to the filament axis. Each Tn is connected to the Tm about 20 nm from one of the Tm ends. Computation of the adjoining molecules by blue and green color emphasizes the two stranded feature of the thin filament with the pitch of 72 nm.

Atomic structure is not taken into consideration because it does not contribute into the low-angle X-ray diffraction spectra; a special attention is drawn to depict the real shape and volume of each of the used elements as well as to correctly arrange all elements within the 3D space. Good agreement in localization of the first seventeen layer-lines (Figure 3) between the calculated and actual diffraction patterns justifies the used simplicity in description of the molecules.

The independent localization of each of the used elements has allowed to simulate three specific muscle states: (1) relaxed, when all myosin heads lie on the filament surface and the actin monomers are axially 2.75 nm apart and angularly at 166.15°; (2) rigor, when all myosin heads are on the surface of surrounding thin filaments and the actin monomers are axially 2.867 nm apart and angularly at 168°; (3) subsequent phases of contraction. Because each myosin molecule is computed from individual subfragments (Figure 1), each of them can be independently localized in the 3D space of the lattice; the independency is limited only by the condition of molecule integrity. The contraction phases are computed by unwrapping of individual S2. Thin filament structure during contraction is computed by introduction of the rotation between the neighboring actin monomers 168° instead of 166.15° and by gradual increasing of the axial distances between five neighboring actin monomers from 2.75 to 2.867 nm.

Each mode of the sarcomere structure is verified by comparison of the calculated Fourier spectrum with actual diffraction patterns available in literature. Fourier transform calculations, described previously [65], are based on the principles of Fresnel’s diffraction and image processing procedure [66].

## 4. Conclusions

On the basis of the obtained results, a new concept of the muscle contraction mechanism is proposed. The concept radically differs from the commonly accepted (reviewed e.g., in [20,23,26,31,37,39,55]) first of all in the assumption that the mutual sliding of the myofilaments is generated not only by the myosin cross-bridges but also by the thin filaments. Second radical difference is applied to the myosin cross-bridge action; each cross-bridge moves from the thick filament surface towards three of six surrounding thin filaments along a helical trajectory, not in an oar-like manner. The movement takes place by cyclical unwrapping and wrapping the thick filament backbone by the S2, probably due to conformational changes within the S2/LMM hinge domain, investigated by Harrington and co-workers [67–69]. In consequence of the strong interactions with myosin heads, the actin filaments gradually elongate towards the sarcomere middle and pull the connected cross-bridges. The mechanism of gradual elongation is shown by animation at site http://sarcomere.ibib.waw.pl. The myosin cross-bridges detach from the actin monomers and come back to the thick filament surface due to a tension arisen within the coiled-coil S2. The cyclical action of the myosin cross-bridges is probably regulated by the C-protein, found on the thick filament surface as occurring with a 43 nm period [70], as well as by the change of electrostatic field around the myofilaments. The conformational change within the myosin head, *i.e*., rotation between the motor and lever-arm domains, taken into consideration in the lever-arm hypothesis [30,31], may strengthen the force.

**Figure 6.**
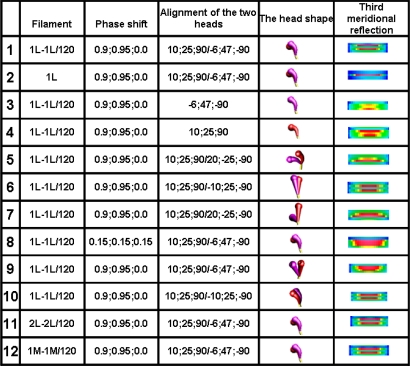
The M3 reflections cut from the Fourier spectra calculated for different geometrical conditions. In the “Filament” column, the configuration of myosin cross-bridge crown and the angle of rotation between the two halves of myosin filament are shown. In the “Phase shift” column, the degrees of unwrapping of the three cross-bridges from three successive crowns are provided. In the “Alignment of the two heads” column, the two sets of three angles are depicted; each set describes the alignment of one of the two heads.

**Figure 7.**
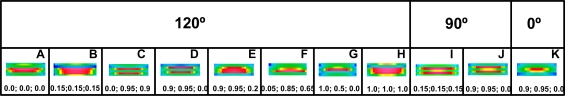
The M3 reflections cut from the Fourier spectra calculated for seven myosin filaments arranged into hexagonal lattice. All myosin filaments have the configuration denoted by 1L-1L. The data are shown for three angles of rotation of the two polar halves: 120°, 90°, and 0°. Under each reflection, the proper phase shift in action of the myosin cross-bridges belonging to three successive crowns is announced.

**Figure 8.**
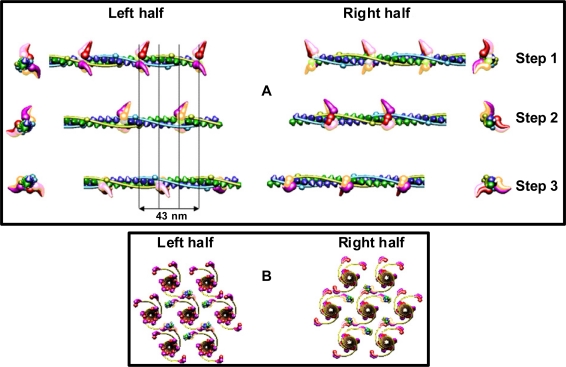
Geometrical conditions needful for the stereo-specific interaction between myosin and actin. The structure of the bipolar hexagonal lattice is the same as that shown in Figure 1. (**A**) The longitudinal and transverse views of the thin filament (shown in (**B**) as the upper thin filament) decorated by myosin heads are shown for three 14.333 nm steps of the filament sliding in the left and right halves of the sarcomere. (**B**) The transverse view of the hexagonal lattice is shown during one of the three steps. Three steps of different arrangement of the myosin cross-bridges exist because the three myosin cross-bridges belonging to the same crown are aligned at angles of 0°, 120°, and 180° and the successive crowns are rotated at 120°. During each step, three myosin cross-bridges belonging to the crowns axially distanced by 43 nm are in the vicinity of the surrounding thin filaments (**B**), but only one of the two myosin heads belonging to the same molecule hits precisely into the proper actin monomer. The connected molecules look as switched on.

The cross-bridge movement along a helical trajectory is more reliable than by bending the myosin molecule within the three hinge domains (see Introduction) because it easily interprets such experimental data as: (1) various perturbations in arrangement of the myosin heads on the filament surface, often observed by EM; (2) the appearance of the “forbidden” reflections in the actual diffraction patterns [44,47,48,56–58] (see Figure 2); (3) the increasing of mass around each thin filament at the line between the neighboring thin filaments [71,72] (see Figure 8B); (4) the stereo-specific interaction between hundreds of binding-sites specific for each myosin head and each actin monomer; (5) the sarcomere contraction at the distances between the two kinds of filaments longer (about 20 nm [73]) or shorter (about 9 nm [74]) than physiological (about 13 nm); (6) the splitting of M3 reflection in transition from relaxed state into contraction [34–36] (see Figures 6, 7); (7) the muscle elasticity observable during contraction (reviewed e.g., in [26,28]). The elasticity should increase during movement along a helical path because the S2 has the structure of two coiled-coil α-helixes [75–77].

The idea of the thin filament elongation is contrary to the commonly accepted view that the F-actin filament helix is a rigid rod. But ability of actin monomers to rotate or recede from one another in the axial direction is usually supported by diffraction data [50,78–80]. Huxley *et al.* [78], Wakabayashi *et al.* [79], and Tsaturyan *et al.* [80] enumerated that the elongation is very small, only about 0.2–0.3%. Bordas *et al.* [50] considered that the elongation can be much larger, up to 1.8–3.2%. The elongation introduced in the work makes 4.25% change, but it seems reliable from the points of view of the good consistency of the obtained results with the experimental data gathered on the basis of literature.

First, the calculated Fourier spectra clearly demonstrate (Figure 2) that increasing of the M15 reflection intensity as well as occurrence of the distinct reflections at the 1st, 2nd, 3rd, and 4th layer lines are possible only if the alignment and the axial distance between the neighboring actin monomers change, respectively, from 166.15° to 168°, and from 2.75 to 2.867 nm. The rotation probably occurs as a result of stimulation by Ca^2+^-ions, and the axial shift in consequence of the strong interaction with myosin head. The values enumerated on the basis of the experimental data could be small because the result depends on a fragment of the elongated thin filament.

Second, it is commonly believed that only the Tm-Tn complex influences the muscle contraction by shielding or exposing the myosin binding-sites on actin monomers, this way enabling or preventing the stereo-specific interaction with myosin head (the hypothesis of steric blocking [10]). The previously performed computer simulation [64] has allowed demonstrating that any displacement of the Tm-Tn complex in relation to the F-actin filament axis without changing the mutual arrangement of actin monomers has no effect on the spectrum.

Third, the new parameters ensure the precise hitting between the specific binding-sites (Figure 8) because each fifth actin monomer is correctly oriented in relation to three surrounding myosin filaments: 24° × 5 = 120°, and 2.8666 × 5 = 14.333 nm. We can estimate that each 14.333-nm shift of the interdigitating filaments towards the sarcomere middle contains the (0.122 × 5)-nm lengthening of the actin filament.

Fourth, the smallest system capable to generate observable movement consists of immobilized head and a fragment of actin filament [81–84].

The computer simulation clearly demonstrates that the hitting needful for the strong actin-myosin interaction is possible only if the coordination in action of myosin cross-bridges exists. The coordination consists of the following: (1) in synchronous action of all thick filaments identically arranged within the hexagonal lattice; (2) in the phase shift in action of the cross-bridges belonging to three successive crowns of the thick filament. Knowledge of the coordination is important for estimation of the force generated by individual cross-bridge on the basis of the force measured for a sarcomere.

More complete description of the new concept of muscle contraction, with consideration of the specific biochemical and biomechanical aspects, will be presented elsewhere.
